# Construction and evaluation of a cloud follow-up platform for gynecological patients receiving chemotherapy

**DOI:** 10.1186/s12913-024-10597-w

**Published:** 2024-01-22

**Authors:** Xin Dan, Ya-Lin He, Yan Huang, Jian-Hua Ren, Dan-Qing Wang, Ru-Tie Yin, Ya-Lin Tian

**Affiliations:** 1 Department of Radiation Therapy and Chemotherapy for Cancer Nursing, West China Second University Hospital, Sichuan University, Chengdu, 610041 Sichuan China; 2 Key Laboratory of Birth Defects and Related Diseases of Women and Children (Sichuan University), Ministry of Education, Chengdu, 610041 Sichuan China; 3 Department of Nursing, West China Second University Hospital, Sichuan University, Chengdu, 610041 Sichuan China; 4 Department of Obstetrics and Gynecology Nursing, West China Second University Hospital, Sichuan University, Chengdu, 610041 Sichuan China; 5 Radiation Therapy and Chemotherapy for Cancer, West China Second University Hospital, Sichuan University, Chengdu, 610041 Sichuan China

**Keywords:** Chemotherapy, Cloud follow-up, Cost-effectiveness, Gynecological malignancy

## Abstract

**Background:**

Patient follow-up is an essential component of hospital management. In the current information era, the patient follow-up scheme is expected to be replaced by Internet technology. This study constructed a cloud follow-up platform for gynecological chemotherapy patients and assessed its cost-effectiveness and patients’ feedback.

**Methods:**

A total of 2,538 patients were followed up using a cloud follow-up system between January and October 2021. Prior to this, 690 patients were followed manually via telephone calls. Patients’ characteristics, follow-up rate, satisfaction, and session duration were compared between the cloud follow-up and manual follow-up groups. In addition, the read rate of health education materials in the cloud follow-up group was analyzed.

**Results:**

General information, including age, education attainment, cancer stage, and disease category, and follow-up rate (cloud: 6,957/7,614, 91.4%; manual: 1,869/2,070, 90.3%; *P* = 0.13) did not significantly differ between the two groups. The follow-up satisfaction of the cloud follow-up patients was significantly better than that of the manual follow-up group (cloud: 7,192/7,614, 94.5%; manual: 1,532/2,070, 74.0%; *P*<0.001). The time spent on the follow-up was approximately 1.2 h for 100 patients in the cloud follow-up group and 10.5 h in the manual follow-up group. Multivariate analysis indicated that the cloud follow-up group had significantly greater follow-up satisfaction (odds ratio: 2.239, 95% CI: 1.237 ~ 5.219). Additionally, the average follow-up duration of the cloud follow-up group decreased by 9.287 h (coefficient: -9.287, 95% CI: -1.439~-0.165). The read rate of health education materials was 72.9% in the cloud follow-up group.

**Conclusions:**

The follow-up effect of the cloud follow-up group was not inferior to that of the manual follow-up group. The cloud follow-up was more effective for prevention and control requirements in the post-epidemic era. Cloud follow-up can save medical resources, improve cost-effectiveness, provide sufficient health education resources for patients, and improve their satisfaction.

## Introduction

Gynecological malignant tumors occur in the female reproductive organs. The most prevalent gynecological cancers are cervical, endometrial, ovarian, fallopian tube, and vulvar cancers, accounting for 19% of new female cancers and seriously threatening women’s health [1]. Chemotherapy is one of the primary means of cancer therapy [2]. It improves the cure rate of cancer and prolongs the long-term survival of cancer patients; however, chemotherapy is associated with several adverse effects [3]. Up to 75% of patients receiving chemotherapy experience chemotherapy-induced nausea and vomiting (CINV) [4]. Even after prophylactic use of antiemetics, such as 5-HT_3_ receptor antagonists, more than 50% of patients present with acute (within 24 h of receiving chemotherapy) or delayed (between 2 and 5 days of treatment) symptoms of CINV [5]. In addition, the incidence of other adverse effects of chemotherapy, such as chemotherapy-induced constipation (CIC), sleep disturbance, chemotherapy-induced peripheral neuropathy (CIPN), and cancer-related fatigue (CRF) is 16–48% [6], 65% [7], 30–40% [8], and 78%, respectively [9]. Typically, these adverse effects do not occur simultaneously, and some of them occur or worsen after discharge [10]. Therefore, it is particularly important to provide regular follow-up and professional health guidance to patients receiving chemotherapy.

Cloud follow-up is a new follow-up mode that uses mobile information technology for continuous nursing. It integrates information technology and medical services. Medical staff sends illustrated medical information to patients through the Internet platform and mutually interact with patients. This mode provides convenience, intelligence, and personalization [11]. Traditional follow-up methods, such as telephone, email, outpatient follow-up, family visits, and community follow-up, require considerable human resources and time. Cloud follow-up can address these deficiencies and help digital management of patients’ information, data processing, and data sharing, thereby saving medical resources and improving the working efficiency of medical staff [12].

Follow-up is an essential and routine aspect of treatment among gynecological patients receiving chemotherapy. In this study, a cloud follow-up platform was constructed for these patients, and cost-effectiveness and patients’ feedback were compared between this follow-up method and the traditional method.

## Methods

### Setting and participants

This study was conducted in a leading maternity and children’s hospital in China. The cloud follow-up platform was introduced into the gynecological tumor chemotherapy ward in 2019 using the hospital information system. In total, 2,538 patients who had undergone chemotherapy for gynecological cancer were enrolled between January and October 2021. This group of patients was defined as the cloud follow-up group since all the follow-up of patients was completed using the cloud follow-up system. In addition, between April and September 2020, 690 patients receiving chemotherapy for gynecological tumors were included in the manual follow-up group. Specifically, patients in this group were followed via telephone calls by nurses.

### Cloud follow-up group

#### Establishment of a multidisciplinary treatment (MDT) team for cloud follow-up

A multidisciplinary treatment (MDT) team with eight members was organized, including one department director, one head nurse, three tumor nurses, one oncologist, and two cloud follow-up information technicians. The department director was primarily responsible for constructing and coordinating the cloud follow-up platform. The head nurse was responsible for formulating a cloud follow-up-related management system and implementing project training. Physicians and nurses answered the medical questions of patients or their family members online from 14:00 to 16:00 every day. Tumor nurses were responsible for establishing disease publicity and education-based knowledge, follow-up from baseline, follow-up rules, etc. The cloud follow-up information technicians were responsible for providing technical support for the information needs of the follow-up of patients with gynecological cancer receiving chemotherapy.

#### Construction of the cloud follow-up platform

The cloud follow-up platform of our hospital was constructed and implemented by a third-party company, jointly managed by the technicians of the third-party company and the information technicians of the hospital. The cloud follow-up platform was mainly developed in Java language and adopted the Apsara technology platform, integrating elastic computing, data storage, CDN storage, and large-scale computing technology. This platform provided storage resources and computing resources to users on the Internet in the form of public services. The cloud follow-up platform included PC-based physician-patient collaboration, a medical App, a patient App, and a WeChat official account. Since July 2020, the cloud follow-up platform has expanded the modules and functions related to the follow-up of gynecological patients receiving chemotherapy (Table 1).

**Table 1:** Modules and functions of cloud follow-up platform

Category	Functional module	Specific functions	Function description
PC-based doctor-patient collaboration platform	Patient management	Patient grouping	After the doctor issues the discharge order, the cloud follow-up platform automatically adds the patient to the cloud follow-up list
		Personality management	Includes health records (such as basic personal information, medical history information, number maintenance, information entry, lifestyle, and laboratory indicators); business records (such as health education records, follow-up form records, short message records, follow-up records, and abnormal monitoring); health records (such as diet records, chemotherapy complications records)
	Knowledge base management	Health education resources	It contains 13 special health education materials for gynecological chemotherapy patients in the form of video, PowerPoint slides, and text.
		Propaganda rule base	Chemotherapy patients started from the day of discharge, followed by a special health education every week on a total of 13 topics.
		Follow-up form resources	Questionnaire on adverse reactions of chemotherapy (including nausea, vomiting, sleep, constipation, diarrhea, deep venous thrombosis, fatigue)
		Follow-up rule base	Follow-up forms will be pushed 2 days, 1 week, and 2 weeks after discharge.
		Reminder phrase resources	Set phrases to remind patients of precautions
	Follow-up center	Follow-up plan	Make a follow-up plan. Medical staff can view the total follow-up personnel, personnel to be followed up, personnel who have been followed up, and overdue follow-up personnel
	Statistical center	Statistical function	Automatically count the reading of each health education theme and the specific filling of each follow-up form and summarize and export the data
Medical App	Health information	Health information	Medical staff can view the patient’s adverse reaction records of chemotherapy online and the patient’s examination report, laboratory test report, follow-up form, etc.
	Doctor-patient interaction	Doctor-patient interaction	Medical staff can monitor the abnormal health information, inspection results, and follow-up results of patients, communicate and interact with patients in real-time, answer patients’ problems in real-time, provide health education and guidance, and realize remote nursing services.
Patient App	Household graded management	Graded management of chemotherapy complications	Graded management is carried out according to the severity of adverse reactions after chemotherapy.
	Consulting service	Consulting service	Provide online interaction for patients. Patients can leave messages in the form of text, pictures, videos, and voice messages or contact medical staff online to solve the problems existing in home care remotely.
	Health monitoring	Medical information	Patients can view medical records, examination reports, and laboratory test reports synchronously through the App.
	Personal settings	Personal settings	Patients can set personal information, medication reminders, follow-up reminders, etc.
WeChat official account	Health education and follow-up		The cloud follow-up platform pushes messages through the open interface of the WeChat public platform. After the patients connected with the WeChat official account, the health education materials and follow-up questionnaires were directly pushed to the main interface of the WeChat official account. Patients used this to acquire knowledge about the disease and fill in the follow-up questionnaires at any time during the daily use of WeChat.

### Implementation of the cloud follow-up platform

#### Establishing patient-specific files

The files included personal basic information (name, gender, age, ID card number, telephone number, etc.) and medical information (current medical problem, past medical history, allergy history, family history, marriage and childbirth history, history of surgery, etc.). It also included outpatient records (medical records, outpatient diagnosis, inspection, examination reports, etc.), inpatient records (admission registration, medical orders, discharge summary, surgical records, hospitalization expenses, examination reports, inspection reports, etc.), and medical examination (medical examination registration and medical examination records).

#### Specialized follow-up and health education

After establishing a specialized file for screening suitable patients, a specialized follow-up pathway was developed. After issuing the discharge order, the cloud follow-up system automatically added the patient to the follow-up list and collected the patient’s basic information. Since most of the patients receiving chemotherapy in the intervention department were discharged the day after the infusion of chemotherapeutic agents, a specific follow-up timeline (2 days, 1 week, and 2 weeks after discharge) was set by the MDT team to investigate the occurrence of acute CINV, delayed CINV, and other chemotherapy complications through the WeChat official account. The follow-up contents were consulted by experts. The items of the follow-up form included adverse reactions after chemotherapy, such as CINV, constipation, diarrhea, fatigue, and sleep disorders. Each symptom contained hidden subquestions, which popped up automatically only when the patient chose to select the symptom. In addition, the system automatically pushed the corresponding health education materials according to the answers provided by the patient. The forms of health education materials included video, PowerPoint, and health education text. Furthermore, the system has set the most severe level for each adverse reaction after chemotherapy. If the patient selected this option, the system assumed that the patient was in a life-threatening state and automatically reminded them to seek medical attention as soon as possible. Subsequently, a report was generated and automatically uploaded to the cloud (Table 2). Medical staff could view the answers filled in by the patients through the medical App and provide necessary feedback. The following Textbox is a simple follow-up dialogue conducted through the WeChat official account.

**Table 2:** Automatically generated table for the dialogue in Table 3

Parameter	Information
Basic information	Patient name, age, diagnosis, registration number, cancer stage, chemotherapy course, and discharge date ^a^
Dialogue result	The patient completed the follow-up form
Follow-up form duration (s)	221
Question 1 score	5
Question 2 score	0
Question 3 score	0
Question 4 score	0
Question 5 score	0
Question 6 score	0
Question 7 score	0
Question 8 score	5
Total score	10
Number of options abnormal	2
Number of times of medical treatment reminded	1
Number of health education materials pushed	1
Follow-up date	2021/5/4

^a^Basic information is automatically extracted from the hospital information management system

**Table 3:** Transcription of a sample dialogue

System: Dear gynecological patients receiving chemotherapy, to promptly understand your condition at home and provide guidance and help, can you spend a few minutes on a follow-up survey?
Patient: OK.
System: Have you experienced nausea in the past 24 h?
Patient: Yes.
The system will automatically pop up related sub-questions about nausea.
System: Please select the degree of nausea you have experienced in the past 24 h.
○Loss of appetite but not reduced food intake
○Reduced food intake without significant weight loss and malnutrition
○Eating significantly decreased, and physical weakness appeared
If the patient selected the third case, the system automatically popped up the red words “Please see a doctor as soon as possible.”
System: Have you vomited in the past 24 h?
Patient: No.
If the patient selected no, the system automatically popped up the next question.
System: Have you had constipation in the past week?
Patient: No.
System: Have you had diarrhea in the past 24 h? (stool is thin and more than three times/day)
Patient: No.
System: Have you felt tired and weak in the past 24 h?
Patient: No.
System: Were you satisfied with the quality of your sleep in the last week?
Patient: Yes
System: Have you had limb swelling, pain, numbness, chest tightness, or polypnea in the past 24 h?
Patient: No.
System: Can you correctly do finger exercises and ankle pump exercise training to prevent thrombosis?
Patient: No.
The system automatically popped up the health education video of finger manipulation and ankle pump movement.
After answering all the questions, click submit.
System: Thank you for your cooperation. We have recorded your problem. Please handle it according to the system prompt, keep the phone unobstructed, and we will follow you if necessary.

#### Household graded management

**Fig. 1:**
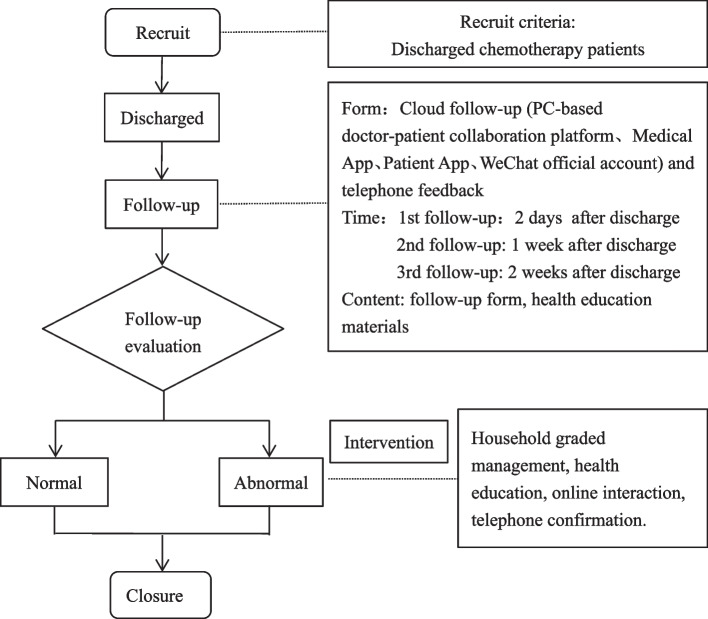
The study flowchart of the cloud follow-up management platform

Patients could record the adverse effects of chemotherapy at home through the patient App, and the medical staff assessed the contents filled in by patients in a real-time mode through the medical App and executed household graded management of patients with abnormal records. In the case of CINV, first-level management was patient self-management. According to the Common Terminology Criteria for Adverse Events v4.0 [13], when nausea and vomiting were rated as grade 0–2, the medical staff conducted one-to-one online guidance through the cloud follow-up system. The second line of management was medical specialty outpatient management. When nausea and vomiting were rated as grade 3–4, the medical staff promptly referred the patients to the hospital’s online system and made confirmation through telephone if necessary. The study flowchart of the cloud follow-up management platform is shown in Fig. 1.

#### Health monitoring

Through the Internet of Things, patients could directly collect health monitoring data from devices such as blood pressure monitors, blood glucose meters, and electrocardiograms. Data were automatically uploaded to the Medical App, and physicians could assess them at any time, achieving continuity between external health data and internal medical data. Particularly, in case of abnormal situations, the system could remind patients according to pre-set reminder rules, and push it to physicians to ensure the safety of patients.

#### Manual follow-up group

The patients in the control group were investigated by manual follow-up. Specifically, nurses contacted patients one by one according to the discharge list of patients. The items of the follow-up form were consistent with those of the cloud follow-up system. However, the uploaded report was filled in manually.

#### Data collection

Seven adverse reactions related to chemotherapy, including nausea and vomiting, constipation, diarrhea, sleep disorders, fatigue, and CIPN, and thrombotic prevention knowledge (finger exercises and ankle pump exercises) were assessed in the two groups of patients on the 2nd day, 1st week, and 2nd week after discharge. The severity of each adverse reaction included 2–5 options. Regarding the design of the options, nausea and vomiting were defined based on the Common Terminology Criteria for Adverse Events v4.0 [13]. Constipation and diarrhea were defined based on the Bristol Stool Form Scale (BSFS) and disease diagnostic criteria [14]. Fatigue was defined based on the Brief Fatigue Inventory (BFI) [15], and sleep quality and CIPN were defined based on the severity of clinical manifestations and their impact on daily life. The mastery of thrombosis prevention knowledge was set to the options of “yes” or “no”. At the last follow-up, follow-up satisfaction was added as an additional item to the questionnaire and divided into five levels: very satisfied, satisfied, average, dissatisfied, and very dissatisfied.

#### Assessment indicators

The assessment indicators were follow-up rate, follow-up satisfaction, session duration, and read rate. The following formulae were used: follow-up rate = number of effective follow-ups / (number of effective follow-ups + number of invalid follow-ups) × 100%; follow-up satisfaction = (number of very satisfied + number of satisfied) / number of total actual follow-up cases × 100%; and read rate = number of read times/number of send times × 100%. The number of effective follow-ups was defined as the complete data collection in Table 2 (excluding the number of health education materials pushed parameter). The number of invalid follow-ups was defined as missing or incomplete data in Table 2. Follow-up satisfaction was defined as patients’ satisfaction with the follow-up service. The number of read times was defined as the total number of patients who actively read health education materials. For the number of send times, we measured how many times the cloud follow-up platform automatically sent health education materials to patients. Session duration was defined as the time medical staff needed to communicate with patients via telephone calls.

#### Statistical analysis

SPSS statistical software (version 22.0, IBM Inc) was used for data analysis. Age, education attainment, cancer stage, disease category, and read rate of health education materials were analyzed descriptively. Patients’ characteristics, follow-up rate, and follow-up satisfaction in both groups were measured using Pearson’s chi-square test. Multiple regression analysis was performed to explore the details of follow-up satisfaction degree and follow-up duration. All tests were two-sided. *P*<0.05 indicated a statistically significant difference.

## Results

**Fig. 2:**
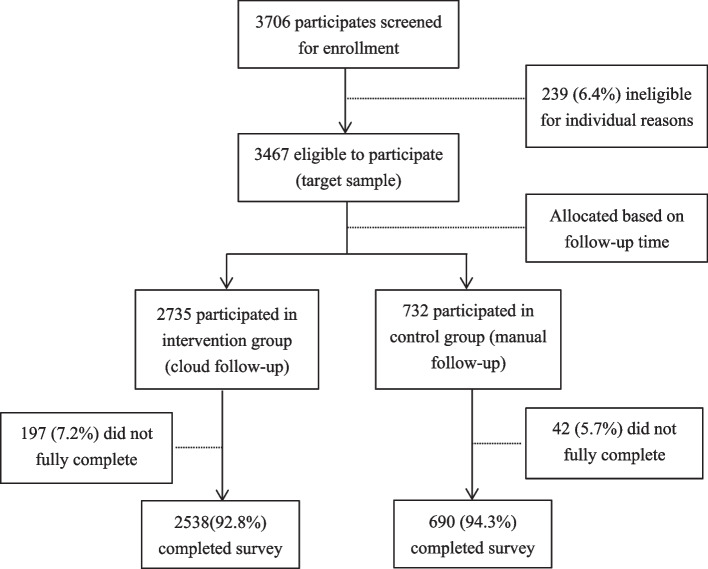
Patient flow

Figure 2 depicts patient recruitment and follow-up processes. In total, 3,706 patients were willing to participate in this study. Among these patients, 239 (6.4%) were excluded due to critical diseases, inability to use smartphones, illiteracy, and poor mental condition. Finally, 3,467 patients were included in this study and were allocated based on follow-up time. Among them, 2,735 patients were assigned to the intervention group for cloud follow-up, whereas 732 patients were assigned to the control group for manual follow-up (telephone follow-up). In total, 197 (7.2%) patients in the cloud follow-up were excluded due to filling out the follow-up form less than three times, while 42 (5.7%) patients in the manual follow-up were excluded due to inability to contact or refusal to participate. Eventually, 2,538 (92.8%) patients in the cloud follow-up group and 690 (94.3%) patients in the manual follow-up group successfully completed the survey.

### Patients’ characteristics

The characteristics of patients in the two groups are shown in Table 4. No significant differences were found between the two groups in age, education attainment, cancer stage, and disease category.

**Table 4:** Comparison of basic information in cloud and manual follow-up group

Characteristics	Cloud follow-up group, n (%)	Manual follow-up group, n (%)	Chi-square (df)	* P* value
**Number of patients**	2538(100)	690(100)		
**Age (years)**			0.54(3)	0.91
<40	337 (13.3)	96(13.9)		
40–49	769 (30.3)	215(31.2)		
50–59	898 (35.4)	236(34.2)		
≥ 60	534 (20.0)	143(20.7)		
**Education**				
<High school	992(39.1)	267(38.7)	0.04(1)	0.85
≥ High school	1546(60.9)	423(61.3)		
**Cancer stage**			3.50(3)	0.32
I	302(11.9)	90(13.0)		
II	783(30.9)	196(28.4)		
III	822(32.4)	214(31.0)		
IV	631(24.9)	190(27.6)		
**Disease category**			2.90(5)	0.72
Cervical carcinoma	822(32.4)	235(34.1)		
Ovarian cancer	655(25.8)	181(26.2)		
Endometrial cancer	700(27.6)	173(25.1)		
Gestational trophoblastic tumor	122(4.8)	39(5.6)		
Vulvar carcinoma	107(4.2)	30(4.4)		
Other	132(5.2)	32(4.6)		

### Cost-effectiveness

Tables 5 and 6 summarize the major outcomes for all participants. When the patients in the two groups completed three follow-ups, the follow-up rate was not significantly different between the two groups (cloud: 6,957/7,614, 91.4%; manual: 1,869/2,070, 90.3%; *P* = 0.13). The follow-up satisfaction degree of cloud follow-up patients was significantly higher than that of manual follow-up group patients (cloud: 7,192/7,614, 94.5%; manual: 1,532/2,070, 74.0%; *P*<0.001). Multivariate logistic regression analysis revealed that cloud follow-up improved patient satisfaction degree (odds ratio: 2.239, 95% CI: 1.237 ~ 5.219). Moreover, 100 patients were randomly selected from the cloud follow-up group to calculate session duration. The total time needed to complete one follow-up was 1.2 h compared with 10.5 h in the manual follow-up group. Multiple linear regression models were applied to assess the adjusted impact of cloud follow-up. The results showed that cloud follow-up significantly reduced follow-up duration. The average difference in follow-up duration was decreased by 9.287 h. In addition, higher education attainment was correlated with lower differences in follow-up duration. Time spent in the manual follow-up group included the time spent by nurses over the telephone to inquire about the patient follow-up form information and arrange and upload Table 2. Table 2 in the cloud follow-up group was automatically and instantaneously generated by the system. The time spent on the cloud follow-up group by medical staff mainly consisted of the time needed to give telephonic feedback on any abnormal form submitted by the patient.

**Table 5:** Comparison of cloud and manual follow-up group indicators

Indicators	Cloud follow-up	Manual follow-up	Chi-square (df)	* P* value
**Follow-up**			2.36 (1)	0.13
Number of effective follow-ups	6957	1869		
Number of invalid follow-ups	657	201		
Follow-up rate, %	91.4	90.3		
**Satisfaction**			764.31(2)	<0.001
Number of very satisfied	4327	887		
Number of satisfied	2865	645		
Number of total actual follow-up cases	7614	2070		
Follow-up service satisfaction, %	94.5	74.0		
**Time spent, hours per 100 patients**	1.2	10.5	N/A^a^	N/A

^a^*N/A* Not applicable

**Table 6:** Regression model for satisfaction and time spent

Variable	Multivariable logistic regression model (Satisfaction)		Multiple linear regression model (Time spent)	
	Odds ratio	95%CI	Coefficient	95%CI
Cloud follow-up (reference group = manual follow-up)	2.239^*^	1.237 ~ 5.219	-9.287^**^	-1.439~-0.165
Age (reference group = age < 40)	0.428	0.287 ~ 1.298	1.021	-0.429 ~ 2.019
Education (reference group = < high school	1.219	0.897 ~ 2.123	-0.876^*^	-0.823~-0.486
Cancer stage (reference group = cancer stage < IV)	0.639	0.329 ~ 1.439	0.927	-0.540 ~ 1.329
Disease category (reference group = cervical carcinoma)	1.232	0.532 ~ 2.987	0.489	-0.829 ~ 2.287
Likelihood ratio test for model	Chi-square = 18.21; *P* = 0.012		Chi-square = 25.23; *P* = 0.008 R squared = 0.311	

^*^P < 0.05; ^**^P < 0.01; Sample size of regression model = 3228

### Pushing and reading thematic health education materials on the cloud follow-up platform

Between January and October 2021, the cloud follow-up platform pushed 170,374 thematic health education materials. Of them, 124,189 were read, with a read rate of 72.9%. Among them, the read rate of “diet, nutrition, and patients receiving chemotherapy” was the highest, followed by “management of nausea and vomiting in patients receiving chemotherapy” and “management of constipation among patients receiving chemotherapy.” “Guidelines for adolescent gynecological patients receiving chemotherapy” had the lowest read rate (Table 7).

**Table 7:** Push and reading of thematic health education materials on cloud follow-up platform

Health education theme	Sending times	Read times	Read rate (%)
Diet, nutrition, and patients receiving chemotherapy	10,152	9827	96.8
Management of nausea and vomiting in patients receiving chemotherapy	20,137	17,357	86.2
Management of constipation in patients receiving chemotherapy	19,827	16,778	84.6
Drug treatment and adverse reactions in patients with gynecological tumors	10,152	8558	84.3
Sleep management of patients receiving chemotherapy	17,998	15,056	83.7
Home care in PICC and infusion port	10,152	7956	78.4
Fatigue management of patients receiving chemotherapy	18,267	14,224	77.9
Management of diarrhea in patients receiving chemotherapy	11,298	7657	67.8
Prevention of thrombosis in patients receiving chemotherapy	10,998	7213	65.6
Diagnosis and management of gynecological tumors	10,672	6987	64.5
Prevention and management of CIPN	11,267	6134	54.4
Fertility related matters of patients receiving chemotherapy	9628	3897	40.5
Guidelines for adolescent gynecological patients receiving chemotherapy	9826	3445	35.1
Total	170,374	124,189	72.9

*PICC* Peripherally inserted central catheters, *CIPN* Chemotherapy induced peripheral neuropathy

### Usage of other functions of the cloud follow-up platform

Of 2,538 patients, 2,212 downloaded the patient App and were registered on it. Patients actively recorded 6,235 diet and nutrition events, 4,256 CINV events, 3218 constipation events, 823 diarrhea events, 3,012 sleep disturbance events, 1,987 peripherally inserted central catheters (PICC) home care events, 924 fatigue events, 328 events of hands and feet numbness, and 1,439 pain events. The automatic data statistics function of the cloud follow-up platform showed that physicians handled abnormal health monitoring data 1,766 times. A medical staff member checked patients’ records through the medical App and provided timely online feedback for abnormal records.

## Discussion

### Cloud follow-up provides a new model for continuous nursing

Continuous nursing aims to extend in-hospital nursing services to communities and families and ensure the continuity of nursing services [16]. Due to the limited length of stay, gynecological patients receiving chemotherapy have different degrees of demand for out-of-hospital nursing services after discharge. Thus, the implementation of continuous nursing for gynecological patients receiving chemotherapy can improve their quality of life (QoL) [17]. In the information era, “Internet +” integrates the Internet and patient follow-up via information and communication technology, forming a modern form of follow-up. Cloud follow-up is one of these products. Cloud follow-up strengthens communication and exchange with patients, promotes the hospital brand, provides more health education resources for patients, and improves treatment compliance. Considering personalized and continuous medical services as the core, we designed efficient information flow and business collaboration channels and built a post-hospital cloud follow-up service system based on the existing service process and functional system of the hospital. This cloud follow-up service system could organically integrate follow-up and clinical practice. Multi-disciplinary cooperation was achieved, helping monitor and manage every patient who used the platform management. This platform also contributed to establishing a comprehensive and accurate medical and health database. With the advancement of science and technology, especially the development of the Internet of Things, cloud follow-up can be combined with other detection devices, making it more convenient and efficient to connect with hospital systems and respond to clinical requirements.

#### Cloud follow-up improved the cost-effectiveness of follow-up

The cost-effectiveness analysis showed no significant difference in the follow-up rate between the cloud follow-up group and the manual follow-up group, suggesting that the follow-up effect of the cloud follow-up group is not inferior to the manual follow-up group and can replace the manual follow-up scheme to a certain extent. The multiple linear regression model showed that the cloud follow-up saved 9.287 h compared with manual follow-up, possibly because the cloud follow-up allowed simultaneous follow-up of 7–9 patients and automatically generated follow-up results. In contrast, in manual follow-up, nurses had to call patients, fill in the follow-up form, and manually generate the follow-up results. Therefore, the cloud follow-up saves time and human resources. In addition, during the follow-up of 100 patients, patients with high school education or above took 0.876 h less time compared with patients with middle school education or below. The majority of patients in this study were middle-aged and elderly. Cloud follow-up, as a new form of follow-up, may be challenging for patients with lower levels of education to understand and use. Patients with higher levels of education are more likely to accept new things and adapt [18]. Even with manual follow-up, patients with lower levels of education may have lower communication and information recognition abilities, resulting in longer follow-up times. Patient satisfaction can objectively reflect the quality of medical services and help measure the quality of hospital management [19]. The multivariable logistic regression model showed that the cloud follow-up group had significantly greater follow-up satisfaction (odds ratio: 2.239, 95% CI: 1.237 ~ 5.219). Cloud follow-up considers medical institutions as the main provider of follow-up services, integrating hospital management systems, physicians, nurses, and researchers to provide patients with more effective post-hospital services. Cloud follow-up combines internet technology with medical and health management for patients, achieving functional requirements such as general follow-up, specialized follow-up, scientific research, questionnaire customization, App interaction, discharge education, and intelligent anomaly analysis. By utilizing the “Internet + Medical”, medical services can be extended to the post-hospital and home settings, enabling patients to receive scientific, professional, and convenient technical services and guidance for rehabilitation and treatment outside the hospital. Moreover, patients in the cloud follow-up group filled in the form or read the health education materials in their spare time. The follow-up time of the cloud follow-up group was more flexible compared with the time needed to cooperate with nurses for manual follow-up. In addition, cloud follow-up can diversely promote health education knowledge via text, PowerPoint, videos, etc., which can make health education funny and enable patients to receive health guidance more intuitively.

### The cloud follow-up platform can reflect patients’ attention to different aspects of disease knowledge

Disease knowledge is closely related to patients’ self-care ability, treatment compliance, and QoL [20–22]. In this study, 13 structured health-education themes were designed based on expert consultation. The read rate of patients exhibited that patients differently paid attention to each theme. Patients paid the highest attention to diet and nutrition, as this part had the most active records. It reflected the knowledge of patients about diet and nutrition and affected their compliance with diet and nutritional modifications, which may be closely related to the prognosis of tumor patients receiving chemotherapy. The 2017 ESPEN guidelines on nutrition in cancer patients recommend enteral nutrition support as a treatment for cancer patients receiving chemotherapy [23]. Therefore, patients pay more attention to diet and nutrition during home care [24]. In addition, patients pay more attention to the management of CINV and constipation [25], since CINV and constipation are the most common adverse effects of chemotherapy and significantly impact patients’ comfort and QoL. Patients pay less attention to fertility-related matters and guidelines for adolescent gynecological patients receiving chemotherapy (read rates were 40.5% and 35.1%, respectively). The reason may be related to the age at which diseases occur. The peak age of gynecological malignant tumors is mainly 40–65 years [26], which is consistent with the results of our study. However, most people in this age group had passed their adolescence and had given birth; therefore, they pay less attention to relevant health education.

### The cloud follow-up platform provided sufficient health education resources for patients and met the requirements of hospital infection management

Gynecological patients receiving chemotherapy have insufficient health education information, especially for home care [27]. However, lack of knowledge is an independent risk factor for treatment non-compliance [28]. The 13 thematic health education materials pushed through the cloud follow-up platform improved the knowledge of gynecological patients receiving chemotherapy. Medical staff regularly logged in to the cloud follow-up platform to check patient click-through rates, assess the active acceptance of health guidance, and provide targeted guidance to patients with low compliance. In addition, the health education materials pushed by the cloud follow-up platform allowed patients or their family members to repeatedly view the education content anytime and anywhere through their mobile phones. The video content, combined with text, images, sound, and animation, makes it easier for patients to remember the treatment of side effects of chemotherapy, thereby improving their QoL. Furthermore, patients in the cloud follow-up platform group more comprehensively consulted medical staff online, obtained professional guidance and medication reminders, and received disease knowledge compared with those receiving manual follow-ups. It has no time and geographical limitations and provides a paradigm for other medical institutions to improve the management of patients without follow-up in remote areas. Information technology provides access to high-quality medical resources, reduces unnecessary outpatient follow-ups, avoids cross-regional patient mobility, and decreases the risk of cross-infection during the COVID-19 pandemic. This approach meets the requirements of hospital infection management.

### Limitations

There are some limitations to the present study. First, in addition to the form of online answers by the medical staff, other forms, such as chatbot, can be introduced. A chatbot is an artificial intelligence program that realizes human-computer interaction in the form of dialogue or text with the help of natural language processing and emotional analysis. It is currently used in the diagnosis, treatment, and management of diseases [29]. It can also help patients with gynecological tumors in solving problems commonly observed in the perioperative period [30]. Therefore, future studies can develop chat machine software or reference software suitable for gynecological patients receiving chemotherapy. Second, the cloud follow-up system used in this study was implemented only in the early stages, including early software testing, system installation training, and hospital pilot. Therefore, clinical operation time was short, and the system was not stable enough. These issues should be addressed in collaboration with a software engineer in the later stages of the operation process.

## Conclusions

This study highlighted that the follow-up effect of the cloud follow-up group was not inferior to that of the manual follow-up group. Cloud follow-up helps COVID-19 prevention and control, improving the cost-effectiveness of follow-up, providing sufficient health education for patients, and reflecting patients’ attention to disease knowledge. Therefore, it can be widely used in clinical practice.

## Data Availability

The data that support the findings of this study are available from the Department of Patient Center, West China Second University Hospital, Sichuan University. However, restrictions apply to the availability of these data, which were used under license for the current study, and so are not publicly available. Data are, however, available from the authors upon reasonable request and with permission of the Department of Patient Center, West China Second University Hospital, Sichuan University.
